# Association of depressive symptoms, physical function, and cardiovascular disease risk in middle-aged and elderly Chinese

**DOI:** 10.3389/fmed.2025.1513614

**Published:** 2025-05-14

**Authors:** Miao Li, Shuo Zhuang, Yan Gao

**Affiliations:** ^1^Department of General Medicine, The 960th Hospital of People's Liberation Army Joint Logistics Support Force, Jinan, China; ^2^First Clinical Medical College, Shandong University of Traditional Chinese Medicine, Jinan, China

**Keywords:** CESD-10, physical function, depressive symptoms, physical dysfunction, cardiovascular disease, China health and retirement longitudinal survey

## Abstract

**Objective:**

Current research suggests that depressive symptoms and physical function increase the risk of developing cardiovascular disease (CVD), but how these factors interact to increase the risk of CVD remains unclear. Therefore, we used data from Chinese middle-aged and older adults to investigate the relationship between depressive symptoms and physical function and CVD risk.

**Methods:**

Using information from the China Health and Retirement Longitudinal Study (CHARLS), we examined the relationship between depressive symptoms, physical functioning, and CVD risk in middle-aged and older Chinese adults. The subsequent seven-year endeavor, which ran from 2011 to 2018, enrolled Chinese adults who were middle-aged and older (≥45 years). The exposures of interest were symptoms of depression and physical impairment. To measure depression symptoms, the Center for Epidemiological Studies Depression Scale (CESD-10) was used. The physical disability was ascertained using the physical mobility function. Its principal endpoint was the incidence of CVD. Cox proportional hazards regression methods has been applied to ascertain 95% of the hazard ratios (HRs) and confidence intervals (CIs). Cox multivariate regression and stratified interaction analysis analyses were employed to investigate the association between depressive symptoms, physical functioning, and CVD.

**Results:**

A total of 1980 people were included, of whom the mean age of the participants was 56.4 ± 7.7 years, of whom 1,013 (51.2%) were women. During the maximum follow-up period of 7 years, 303 (15.3%) suffered from cardiovascular disease, of whom 246 (12.4%) had heart disease and 72 (3.6%) suffered from stroke. Compared with those with NDS (no depressive symptoms) (CESD <10) and NPD (no physical dysfunction), those with both DS (depressive symptoms) (CESD ≥10) and PD (physical dysfunction) had the highest risk of overall CVD (adjusted hazard ratio [HR], 1.88; 95% CI 1.18 to 3), coronary heart disease (HR, 2.45; 95% CI 1.44 to 4.18) and stroke (HR, 0.45; 95% CI 0.15 to 1.31), which were most common in people aged 60 years or younger.

**Conclusion:**

This study found that older adults with DS and PD were strongly associated with an increased risk of CVD.

## Introduction

Cardiovascular disease has become increasingly common in recent years. According to a report issued by the International Cardiovascular Disease Federation, 12.57% of people worldwide would have cardiovascular disease by the year 2,045. Heart disease is thought to be a serious public health issue that affects millions of people worldwide and lowers survival rates. Since 2006, the prevalence of cardiovascular disease (CVD) in China has grown. In 2018, there were an estimated 290 million persons with CVD. In terms of mortality, CVD accounts for more than half of all fatalities in China—more than cancer and all other illnesses combined. Therefore, early detection of cardiovascular disease risks offers a chance to prevent or postpone the beginning of the CVD (1).

We further searched for data on the prevalence of major depression and found that the prevalence of depression in the elderly population was already as high as 13.3 per cent prior to the New Crown epidemic and increased to 19.3% during the global epidemic (2). In China, women are more likely to be depressed than men, the unemployed are more likely to be depressed than the employed, and those who are separated, widowed or divorced are up to 13% more likely to be depressed than those who are married or cohabiting (3). Research has indicated that depressive symptoms are frequently present in individuals with CVD, and there is evidence that depressive symptoms are associated with a higher risk of developing CVD in adult Chinese (4). Established risk factors for depressive symptoms include prior depressive episodes, physical dysfunction, cognitive impairment, and female gender. Importantly, physical dysfunction may not only result from depression but also independently predict its onset (5). It has additionally been demonstrated that a number of cardiovascular disease risk variables have an advantageous relationship with physical dysfunction, and that physical dysfunction have a positive correlation with cardiovascular disease (6, 7).

DS was negatively associated with PD, especially in the elderly CVD cohort, where steps decreased by 1.2 and 2.7% per year of age, respectively. Despite this, the study had limitations as it only covered step counts and British men (8). There is evidence that physical dysfunction is significantly connected with depressive symptoms in middle-aged and older Chinese people (5). However, there are no articles on the association of DS, PD and cardiovascular disease in Chinese middle-aged and elderly people, and compared with the previous literature that considered only lower limb mobility (8), we used a more comprehensive approach to assess PD, which included a thorough assessment of upper and lower limb strength. Based on numerous prospective data collected from respondents across China through the CHARLS, this study aims to investigate the risk factors for cardiovascular disease in middle-aged and elderly individuals in China. Our study’s data was gathered from a sample of people aged 45 and older. We concentrated on evaluating the connection between depressive symptoms, physical functioning, and CVD in Chinese middle-aged and older individuals based on the aforementioned data.

## Methods

### Study design and participants

The is a longitudinal study. The data come from the national survey data of CHARLS in 2011 and 2018. The project was carried out and implemented by the National Development Research Institute of Peking University to conduct household surveys on middle-aged and elderly people (45+) in China. The baseline survey of the CHARLS involved 17,708 people who participated in 2011. The CHARLS survey project was approved by the Biomedical Ethics Committee of Peking University, and all participants were required to sign informed consent. For more information, please visit http://charls.pku.edu.cn.

**Figure 1:**
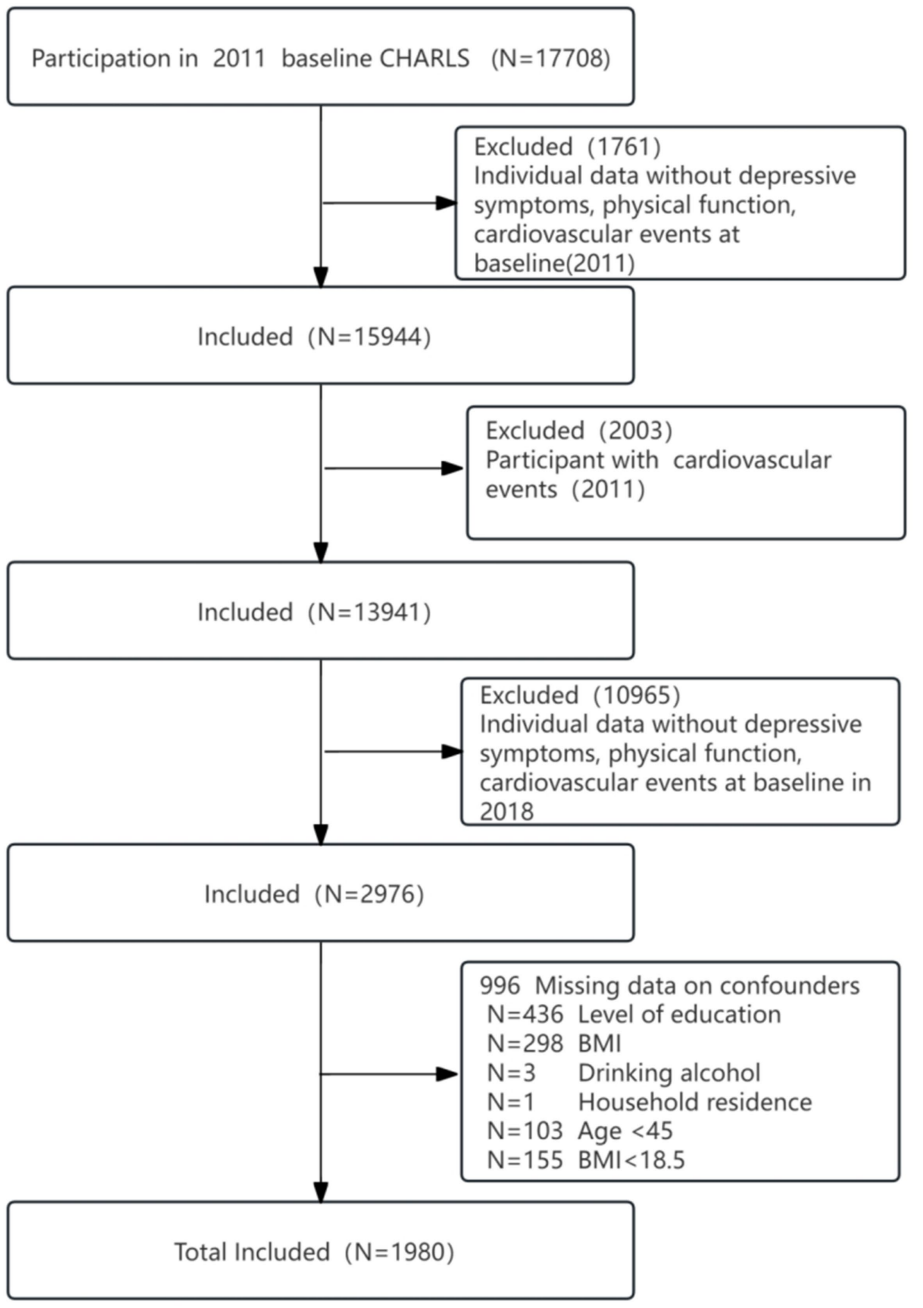
Flowchart of the screening process for the selection of eligible participants.

The CHARLS baseline survey included 17,596 respondents, followed by subject exclusion criteria:(1), missing data on depressive symptoms, physical dysfunction, and cardiovascular events in 2011 (*n* = 1761); (2), participants with a cardiovascular event in 2011 (*n* = 2003); (3), depressive symptoms, physical dysfunction, and cardiovascular events in 2018 were missing (*n* = 10,965); and (4), missing data on age <45 years, body mass index (BMI) < 18.5, and education, BMI, alcohol consumption, and home residence (*n* = 996). Therefore, we ultimately included 1980 participants in the analyses (Figure 1).

### Depressive

The Epidemiological Studies Center the CHARLS questionnaire’s Depression Scale (CESD-10) was utilized to gage depressive symptoms. The CESD-10 demonstrated exceptional reliability and efficacy in accurately assessing depression symptoms in middle-aged and older individuals (9). In accordance with the study, Chinese adults with a CESD-10 score of 10 had respectable specificity (0.80) and sensitivity (0.85) (10). Ten questions make up the CDES-10, and the answers range from 0 to 3. The alternatives are “rarely or none of the time (< 1 day), some or a few times (1–2 days), occasionally or moderately (3–4 days), most of the time (5–7 days).” The documentary determined the ultimate score. More severe depressive symptoms are indicated by higher scores; “no depressive symptoms” (NDS) are confirmed by scores of 10 or fewer inches, while “depressive symptoms” (DS) are indicated by scores above 10 (11).

### Physical function

The CHARLS questionnaire asks about a variety of physical functioning activities. These include: walking 100 meters, jogging or running 1 km, wandering 1 km, picking up a small coin from the table, sitting in a chair for a long time and then getting up, climbing multiple floors repeatedly, bending over, bending knees, or squatting, stretching arms along your shoulders, and running or jogging 1 km. Each question was answered in four different ways. (1) Not at all difficult; (2) challenging but yet doable; (3) challenging and requiring assistance; and (4) not able to finish. If a subject reported difficulty with any of the 9 items, they were defined as having a physical dysfunction (PD), patients were defined as having no physical dysfunction (NPD) if they had no difficulty with each item (12).

### CVD

The study outcomes were CVD events, including heart attack and stroke. Similar to previous studies (29), CVD events were assessed by the following question: Has your doctor ever told you that you have been diagnosed with heart disease, angina, coronary artery disease, heart failure, or other heart problems? Participants who reported having a heart attack or stroke were defined as having CVD. In the 2018 CHARLS questionnaire, CVD was assessed by the following question: Has a doctor ever told you that you have been diagnosed with heart disease, angina, coronary artery disease, heart failure or other heart problems?’ Participants who reported having a heart attack or stroke were defined as having CVD.

### Sociodemographic characteristics, lifestyle behaviors, and long-term conditions

At baseline (2011), trained interviewers used a structured questionnaire to collect information on socio demographic situation and health-related factors, including age, sex, social activities, and educational attainment. Educational achievement is classified as junior high school and below, or senior high school and above (high school, university, postgraduate, doctoral). Health-related factors included self-reported smoking and drinking status (none or yes), and self-reported physician-diagnosed diseases (diabetes, hypertension, heart disease, and dyslipidemia). Laboratory tests included blood urea nitrogen (BUN), C-reactive protein (CRP), uric acid (UA), and Cystatin-C. Marital status was divided into two categories: married and other marital status (married, but not living with the spouse for work, separated, divorced, widowed or never married). Diabetes mellitus was defined as fasting plasma glucose greater than 126 mg/dL (converted to mmol/L and multiplied by 0.0555) and a self-reported history of diabetes. Hypertension was defined as systolic blood pressure greater than 140 mm Hg or diastolic blood pressure greater than 90 mm Hg. Dyslipidemia was defined as total cholesterol >240 mg/dL (converted to mmol/L and multiplied by 0.0259), triglycerides >150 mg/dL, low-density lipoprotein cholesterol (LDL-C) cholesterol >160 mg/dL, high-density lipoprotein cholesterol (HDL-C) cholesterol <40 mg/dL, or a self-reported history of dyslipidemia.

### Statistical analysis

Data are described as the means and standard deviation (SD) for continuous variables. Data that do not fit a normal distribution are described as median. Frequency with percentage was used to describe categorical variables. Baseline characteristics are summarized according to joint assessments of the CESD (10 as cutoff point) and PD (yes or no) and compared among participants of four groups (NDS & NPD, NDS & PD, DS & NPD and DS & PD) using the *χ*^2^ test or analysis of variance, as appropriate.

The multivariate Cox proportional hazards regression model is used to estimate the relationship between depressive symptoms, physical dysfunction, and CVD. Three Cox regression models have been constructed: Model 1 was modified for sex and age; the second model further adjusted for social activities, BMI, education, smoking and alcohol consumption; Model 3 further adjusted for hypertension, dyslipidemia, diabetes mellitus, CysC, CRP, UA.

In addition to the separate and joint analyses of depressive symptoms and physical functioning with CVD risk, we sought to conduct subgroup analyses of the separate and joint associations of depressive symptoms and physical functioning with CVD risk. Subgroup analyses were conducted according to the following selection criteria: age (45–60 or ≥60 years) and hypertension (yes or no). Because there were missing values for educational attainment and body mass index (BMI) in CHARLS and they were only used as covariates, we interpolated the missing data resulting from lower secondary school education and below (*n* = 436) and the use of an average body mass index (*n* = 298). In addition, we performed sensitivity analyses on the interpolated sensitivity study population (*n* = 2,297) (Figure 1).

Statistical analyses were performed using FreeStatisticsV1.9.2^1^. We estimated the association between DS and PD and CVD risk using Cox regression models with HRs and 95% CIs, with year as the underlying time frame. A threshold of 0.05 was established for statistical significance.

## Results

### Baseline characteristics

The baseline characteristics of the study participants in terms of DS and PD are presented in Table 1. Of these, 75.4% had PD, 36.82% had DS and CESD-10 scores ≥10, and 29.19% had both. The average age of the participants in 2011 was 56.4 years [standard deviation (SD) = 7.7], and was higher among those with physical impairment (57.2 ± 8.2; 57.6 ± 7.6). PD was more likely in married individuals with diabetes, dyslipidemia or hypertension. At the same time, they were less involved in social activities (51.3%), most of them were from rural areas (86.5%), and 90.8% had only junior high school or less than junior high school education (Table 1), which was higher in the group with PD. Among the drinking population, there are more people with physical impairments than those without. Lower values for creatinine, uric acid and hgb (0.7 ± 0.2) were found in the group with both DS and PD; however, higher values for total cholesterol, HDL, triglycerides and C-reactive protein were found in the group with both DS and PD (Table 1).

**Table 1:** Baseline characteristics of the study population cross-classified by depressive symptoms and physical functioning.

Variables	Total (*n* = 1980)	NDS + NPD (45/580) (*n* = 580)	NDS + PD (*n* = 671)	DS + NPD (*n* = 151)	DS + PD (*n* = 578)	*p*	Statistic
Age	56.4 ± 7.7	54.7 ± 6.9	57.2 ± 8.2	54.7 ± 7.2	57.6 ± 7.6	< 0.001	19.141
Sex						< 0.001	114.208
Male	967 (48.8)	379 (65.3)	297 (44.3)	86 (57)	205 (35.5)		
Female	1,013 (51.2)	201 (34.7)	374 (55.7)	65 (43)	373 (64.5)		
Marital status						0.182	4.865
Other marital status	97 (4.9)	20 (3.4)	33 (4.9)	8 (5.3)	36 (6.2)		
Married	1883 (95.1)	560 (96.6)	638 (95.1)	143 (94.7)	542 (93.8)		
Hypertension						< 0.001	24.026
No	1,632 (82.4)	513 (88.4)	546 (81.4)	124 (82.1)	449 (77.7)		
Yes	348 (17.6)	67 (11.6)	125 (18.6)	27 (17.9)	129 (22.3)		
Dyslipidemia						0.245	4.156
No	1872 (94.5)	557 (96)	627 (93.4)	143 (94.7)	545 (94.3)		
Yes	108 (5.5)	23 (4)	44 (6.6)	8 (5.3)	33 (5.7)		
Diabetes						0.006	12.317
No	1911 (96.5)	572 (98.6)	641 (95.5)	147 (97.4)	551 (95.3)		
Yes	69 (3.5)	8 (1.4)	30 (4.5)	4 (2.6)	27 (4.7)		
Social contact						< 0.001	16.518
No	1,016 (51.3)	269 (46.4)	331 (49.3)	84 (55.6)	332 (57.4)		
Yes	964 (48.7)	311 (53.6)	340 (50.7)	67 (44.4)	246 (42.6)		
BMI (kg/m^2^)						0.025	14.496
18.5–23.9	1,232 (62.2)	373 (64.3)	390 (58.1)	107 (70.9)	362 (62.6)		
24–27.9	561 (28.3)	163 (28.1)	203 (30.3)	36 (23.8)	159 (27.5)		
≥28	187 (9.4)	44 (7.6)	78 (11.6)	8 (5.3)	57 (9.9)		
Smoke						< 0.001	70.13
No	1,207 (61.0)	281 (48.4)	432 (64.4)	82 (54.3)	412 (71.3)		
Yes	773 (39.0)	299 (51.6)	239 (35.6)	69 (45.7)	166 (28.7)		
Drinking						< 0.001	32.477
No	1,148 (58.0)	390 (67.2)	367 (54.7)	91 (60.3)	300 (51.9)		
Yes	832 (42.0)	190 (32.8)	304 (45.3)	60 (39.7)	278 (48.1)		
Educational attainment						0.001	16.252
Junior high school and below	1798 (90.8)	511 (88.1)	602 (89.7)	138 (91.4)	547 (94.6)		
High school and above	182 (9.2)	69 (11.9)	69 (10.3)	13 (8.6)	31 (5.4)		
Place of residence						< 0.001	38.419
Urban	267 (13.5)	113 (19.5)	87 (13)	25 (16.6)	42 (7.3)		
Rural	1713 (86.5)	467 (80.5)	584 (87)	126 (83.4)	536 (92.7)		
BUN (mmol/L)	15.8 ± 4.7	15.6 ± 4.6	15.9 ± 4.9	16.1 ± 4.1	15.8 ± 4.7	0.76	0.391
CREA (mg/dL)	0.8 ± 0.2	0.8 ± 0.2	0.8 ± 0.2	0.8 ± 0.2	0.7 ± 0.2	< 0.001	13.645
Cys-C (mg/L)	1.0 ± 0.2	1.0 ± 0.2	1.0 ± 0.2	1.0 ± 0.2	1.0 ± 0.2	0.189	1.595
CHO (mg/dL)	193.6 ± 37.8	193.1 ± 37.4	193.2 ± 37.7	194.1 ± 36.6	194.2 ± 38.7	0.964	0.092
HDL (mg/dL)	51.2 ± 14.6	50.4 ± 13.7	51.2 ± 14.8	50.3 ± 13.7	52.3 ± 15.4	0.246	1.383
LDL (mg/dL)	114.8 ± 33.5	115.0 ± 35.5	114.1 ± 31.8	116.2 ± 36.8	115.0 ± 32.7	0.926	0.156
TG (mg/dL)	103.5 (73.5, 147.8)	100.4 (71.7, 147.8)	102.7 (72.6, 144.5)	100.0 (77.0, 150.4)	106.2 (77.0, 147.8)	0.623	1.763
CRP (mg/L)	2.7 ± 8.7	2.6 ± 10.1	2.6 ± 5.7	1.9 ± 3.6	3.2 ± 10.7	0.427	0.928
UA (mg/dL)	4.5 ± 1.2	4.7 ± 1.2	4.5 ± 1.2	4.5 ± 1.3	4.3 ± 1.1	< 0.001	9.919
HGB (g/dL)	14.3 ± 2.4	14.6 ± 2.3	14.2 ± 2.5	14.6 ± 2.6	14.0 ± 2.4	< 0.001	5.476
HBA1C (%)	5.3 ± 0.7	5.2 ± 0.6	5.3 ± 0.7	5.3 ± 0.9	5.3 ± 0.8	0.016	3.465

BMI, body mass index (calculated as weight in kilograms divided by height in meters squared), BUN (blood urea nitrogen), CREA (creatinine), Cys-C (Cystatin-C), CHO (total cholesterol), HDL (High density lipoprotein cholesterol), LDL (Low Density Lipoprotein), TG (triglycerides), CRP (C-reaction protein), UA (Uric acid), HGB (hemoglobin), HbA1c (Glycated haemoglobins).

### Multivariate cox proportional models for joint association of depressive symptoms and physical dysfunction with cardiovascular outcome events

As shown in Table 2, Cox proportional hazard analysis revealed a significant association between the CESD and CVD in the model 1 [HR (95% CI), 1.03 (1.01–1.04), *p* < 0.05]. And the model 2 [HR (95% CI), 1.03 (1.01–1.05), *p* < 0.05], model 3 [HR (95% CI), 1.04 (1.01–1.06), *p* < 0.05] when the CESD was considered as a continuous variable. In contrast with group with CESD<10, The HR for CVD in group with CESD≥10 was 1.58 (95% CI, 1.15–2.16) after adjusting for confounding covariates. The physical dysfunction group had a higher risk of CVD compared with the non-physical dysfunction group (model 1 HR 1.6, 95% CI 1.23–2.08; model 2 HR 1.64, 95% CI 1.25–2.14; model 3 HR 1.61, 95% CI 1.11–2.34) (Table 2). After interpolating the missing values, the sensitivity analysis is consistent with the results in Table 2 (Table 3).

**Table 2:** Association of depressive symptoms and physical functioning with prevalence of CVD

Variables	Model 1	adj. *p* value	Model 2	adj. *p* value	Model 3	adj. *p* value
CESD	1.03 (1.01~1.04)	0.005	1.03 (1.01~1.05)	0.003	1.04 (1.01~1.06)	0.005
CESD<10	1(Ref)		1(Ref)		1(Ref)	
CESD≥10	1.43 (1.14~1.8)	0.002	1.47 (1.17~1.85)	0.001	1.58 (1.15~2.16)	0.005
Physical Dysfunction						
No	1(Ref)		1(Ref)		1(Ref)	
Yes	1.6 (1.23~2.08)	0.001	1.64 (1.25~2.14)	<0.001	1.61 (1.11~2.34)	0.013

Model 1: Adjusted for age (45-60/≥60), sex (male/female)

Model 2: Multivariate model adjusted for education (junior high school and below/senior high school and above), smoking (yes/no), alcohol consumption (yes/no), BMI (Body Mass Index), social activities (yes/no)

Model 3: Adjusted for hypertension (yes/no), dyslipidaemia (yes/no), diabetes mellitus (yes/no), CysC (Cystatin-C), CRP (C-reaction protein), UA (Uric acid).

**Table 3:** Baseline characteristics of the study population cross-classified by depressive symptoms and physical functioning (interpolated).

Variables	Total (*n* = 2,297)	NDS + NPD (45/580) (*n* = 651)	NDS + PD (*n* = 771)	DS + NPD (*n* = 180)	DS + PD (*n* = 695)	*p*	Statistic
Age	57.1 ± 8.1	55.2 ± 7.2	58.0 ± 8.6	55.5 ± 7.9	58.4 ± 8.1	< 0.001	23.935
Sex						< 0.001	123.596
Male	1,076 (46.8)	410 (63)	328 (42.5)	100 (55.6)	238 (34.2)		
Female	1,221 (53.2)	241 (37)	443 (57.5)	80 (44.4)	457 (65.8)		
Marital status						< 0.001	21.441
Never married	368 (16.0)	74 (11.4)	119 (15.4)	33 (18.3)	142 (20.4)		
Married	1929 (84.0)	577 (88.6)	652 (84.6)	147 (81.7)	553 (79.6)		
Hypertension						< 0.001	28.047
No	1885 (82.1)	576 (88.5)	620 (80.4)	148 (82.2)	541 (77.8)		
Yes	412 (17.9)	75 (11.5)	151 (19.6)	32 (17.8)	154 (22.2)		
Dyslipidemia						0.133	5.597
No	2,169 (94.4)	626 (96.2)	720 (93.4)	170 (94.4)	653 (94)		
Yes	128 (5.6)	25 (3.8)	51 (6.6)	10 (5.6)	42 (6)		
Diabetes						0.016	10.328
No	2,215 (96.4)	639 (98.2)	737 (95.6)	176 (97.8)	663 (95.4)		
Yes	82 (3.6)	12 (1.8)	34 (4.4)	4 (2.2)	32 (4.6)		
Social contact						< 0.001	19.356
No	1,169 (50.9)	293 (45)	384 (49.8)	101 (56.1)	391 (56.3)		
Yes	1,128 (49.1)	358 (55)	387 (50.2)	79 (43.9)	304 (43.7)		
BMI						0.006	18.292
18.5–23.9	1,434 (62.4)	420 (64.5)	446 (57.8)	128 (71.1)	440 (63.3)		
24–27.9	641 (27.9)	179 (27.5)	233 (30.2)	43 (23.9)	186 (26.8)		
≥28	222 (9.7)	52 (8)	92 (11.9)	9 (5)	69 (9.9)		
Smoke						< 0.001	75.4
No	1,439 (62.6)	329 (50.5)	508 (65.9)	100 (55.6)	502 (72.2)		
Yes	858 (37.4)	322 (49.5)	263 (34.1)	80 (44.4)	193 (27.8)		
Drinking						< 0.001	32.572
No	1,150 (58.0)	392 (67.2)	367 (54.7)	91 (60.3)	300 (51.9)		
Yes	833 (42.0)	191 (32.8)	304 (45.3)	60 (39.7)	278 (48.1)		
Educational attainment						< 0.001	19.046
Junior high school and below	2,115 (92.1)	582 (89.4)	702 (91.1)	167 (92.8)	664 (95.5)		
High school and above	182 (7.9)	69 (10.6)	69 (8.9)	13 (7.2)	31 (4.5)		
Place of residence						< 0.001	43.155
Urban	325 (14.2)	133 (20.5)	107 (13.9)	29 (16.1)	56 (8.1)		
Rural	1971 (85.8)	517 (79.5)	664 (86.1)	151 (83.9)	639 (91.9)		
BUN (mmol/L)	15.8 ± 4.7	15.6 ± 4.5	15.9 ± 4.9	16.0 ± 4.2	15.8 ± 4.7	0.64	0.562
CREA (mg/dL)	0.8 ± 0.2	0.8 ± 0.2	0.8 ± 0.2	0.8 ± 0.2	0.7 ± 0.2	< 0.001	13.806
Cys-C (mg/L)	1.0 ± 0.2	1.0 ± 0.2	1.0 ± 0.2	1.0 ± 0.2	1.0 ± 0.2	0.18	1.634
CHO (mg/dL)	194.1 ± 38.5	192.8 ± 36.9	193.9 ± 38.1	196.1 ± 37.7	194.9 ± 40.5	0.753	0.4
Hdl (mg/dL)	51.5 ± 14.8	50.7 ± 13.9	51.3 ± 15.0	51.5 ± 14.2	52.4 ± 15.4	0.276	1.289
Ldl (mg/dL)	115.3 ± 34.3	114.9 ± 35.1	115.0 ± 32.7	118.3 ± 37.3	115.5 ± 34.5	0.771	0.376
TG (mmol/L)	102.7 (73.5, 146.0)	99.1 (71.2, 147.8)	103.1 (72.6, 144.9)	99.1 (74.3, 146.9)	104.4 (76.6, 146.0)	0.716	1.356
CRP (mg/L)	2.7 ± 8.4	2.7 ± 9.9	2.5 ± 5.4	2.2 ± 4.8	3.1 ± 10.0	0.545	0.712
UA (mg/dL)	4.5 ± 1.2	4.7 ± 1.2	4.5 ± 1.2	4.4 ± 1.3	4.3 ± 1.1	< 0.001	10.276
HGB	14.3 ± 2.4	14.6 ± 2.3	14.2 ± 2.5	14.6 ± 2.7	14.0 ± 2.4	< 0.001	5.855
HbA1c	5.3 ± 0.7	5.2 ± 0.6	5.3 ± 0.7	5.2 ± 0.9	5.3 ± 0.8	0.007	4.104

BMI, body mass index (calculated as weight in kilograms divided by height in meters squared), BUN (blood urea nitrogen), CREA (creatinine), Cys-C (Cystatin-C), CHO (total cholesterol), HDL (High density lipoprotein cholesterol), LDL (Low Density Lipoprotein), TG (triglycerides), CRP (C-reaction protein), UA (Uric acid), HGB (hemoglobin), HbA1c (Glycated haemoglobins).

### Depressive symptoms, physical dysfunction and cardiovascular outcome events

Compared with those with NDS + NPD, those with both DS and PD had the highest risk of overall CVD in the model 1 [HR (95% CI), 1.86 (1.35 ~ 2.58), *p* < 0.001; HR (95% CI), 2.14 (1.48 ~ 3.07), *p* < 0.001; HR (95% CI), 0.88 (0.44 ~ 1.73), *p* = 0.706], And after adjusting for covariates, Model 2 [HR (95% CI), 2.08 (1.5 ~ 2.9), *p* < 0.001; HR (95% CI), 2.44 (1.68 ~ 3.5), *p* < 0.001; HR (95% CI), 0.93 (0.47 ~ 1.87), *p* = 0.843] and Model 3 [HR (95% CI), 1.95 (1.25 ~ 3.07), *p* < 0.05; HR (95% CI), 2.45 (1.44 ~ 4.18), *p* < 0.05; HR (95% CI), 0.45 (0.15 ~ 1.31), *p* = 0.143]. Due to the small number of stroke positives, we considered it to be clinically irrelevant (Table 4). The results remained consistent after sensitivity analyses were performed (Table 5).

**Table 4:** Combined association of depressive symptoms and physical functioning with prevalence of CVD.

Variables	Model 1		Model 2		Model 3	
	HR (95% CI)	adj. *p* value	HR (95% CI)	adj. *p* value	HR (95% CI)	adj. *p* value
CVD (cases/person-years)						
NDS + NPD (59/4060)	1 (Ref)		1 (Ref)		1 (Ref)	
NDS + PD (107/4697)	1.44 (1.04 ~ 2)	0.027	1.5 (1.08 ~ 2.08)	0.015	1.24 (0.78 ~ 1.96)	0.368
DS + NPD (19/1057)	1.24 (0.74 ~ 2.07)	0.424	1.33 (0.79 ~ 2.24)	0.278	1.06 (0.5 ~ 2.24)	0.885
DS + PD (118/4046)	1.86 (1.35 ~ 2.58)	<0.001	2.08 (1.5 ~ 2.9)	<0.001	1.95 (1.25 ~ 3.07)	0.004
CHD (cases/person-years)						
NDS + NPD (45/4060)	1 (Ref)		1 (Ref)		1 (Ref)	
NDS + PD (83/4697)	1.47 (1.01 ~ 2.13)	0.042	1.54 (1.06 ~ 2.24)	0.023	1.43 (0.83 ~ 2.46)	0.202
DS + NPD (15/1057)	1.27 (0.71 ~ 2.27)	0.427	1.4 (0.78 ~ 2.51)	0.266	1.32 (0.56 ~ 3.14)	0.526
DS + PD (103/4046)	2.14 (1.48 ~ 3.07)	<0.001	2.44 (1.68 ~ 3.54)	<0.001	2.45 (1.44 ~ 4.18)	0.001
Stroke (cases/person-years)						
NDS + NPD (17/4060)	1 (Ref)		1 (Ref)		1 (Ref)	
NDS + PD (31/4697)	1.28 (0.69 ~ 2.35)	0.43	1.31 (0.71 ~ 2.42)	0.384	0.8 (0.31 ~ 2.04)	0.642
DS + NPD (5/1057)	1.1 (0.4 ~ 2.98)	0.853	1.12 (0.41 ~ 3.05)	0.825	0.98 (0.25 ~ 3.84)	0.98
DS + PD (19/4046)	0.88 (0.44 ~ 1.73)	0.706	0.93 (0.47 ~ 1.87)	0.843	0.45 (0.15 ~ 1.31)	0.143

Model 1: Adjusted for age (45–60/≥60), sex (male/female).

Model 2: Multivariate model adjusted for education (junior high school and below/senior high school and above), smoking (yes/no), alcohol consumption (yes/no), BMI (Body Mass Index), social activities (yes/no).

Model 3: Adjusted for hypertension (yes/no), dyslipidaemia (yes/no), diabetes mellitus (yes/no), CysC (Cystatin-C), CRP (C-reaction protein), UA (Uric acid).

**Table 5:** Association of depressive symptoms and physical functioning with prevalence of CVD (interpolated).

Variables	Model 1	adj. *p* value	Model 2	adj. *p* value	Model 3	adj. *p* value
CESD	1.02 (1.01 ~ 1.04)	0.003	1.03 (1.01 ~ 1.05)	0.002	1.04 (1.01 ~ 1.06)	0.004
CESD<10	1 (Ref)		1 (Ref)		1 (Ref)	
CESD≥10	1.39 (1.12 ~ 1.71)	0.002	1.47 (1.17 ~ 1.86)	0.001	1.54 (1.11 ~ 2.13)	0.01
Physical dysfunction						
No	1 (Ref)		1 (Ref)		1 (Ref)	
Yes	1.76 (1.37 ~ 2.26)	<0.001	1.59 (1.21 ~ 2.09)	0.001	1.44 (0.98 ~ 2.13)	0.065

Model 1: Adjusted for age (45–60/≥60), sex (male/female).

Model 2: Multivariate model adjusted for education (junior high school and below/senior high school and above), residence (urban/rural), smoking (yes/no), alcohol consumption (yes/no), BMI (Body Mass Index), marriage (yes/no), social activities (yes/no).

Model 3: Adjusted for hypertension (yes/no), dyslipidaemia (yes/no), diabetes mellitus (yes/no), and BUN (blood urea nitrogen), CREA (creatinine), CysC (Cystatin-C), CHO (total cholesterol), HDL (High density lipoprotein cholesterol), LDL (Low Density Lipoprotein), TG (triglycerides), CRP (C-reaction protein), UA (Uric acid), HGB (hemoglobin), HbA1c (Glycated haemoglobins).

### Subgroup analysis

**Figure 2:**
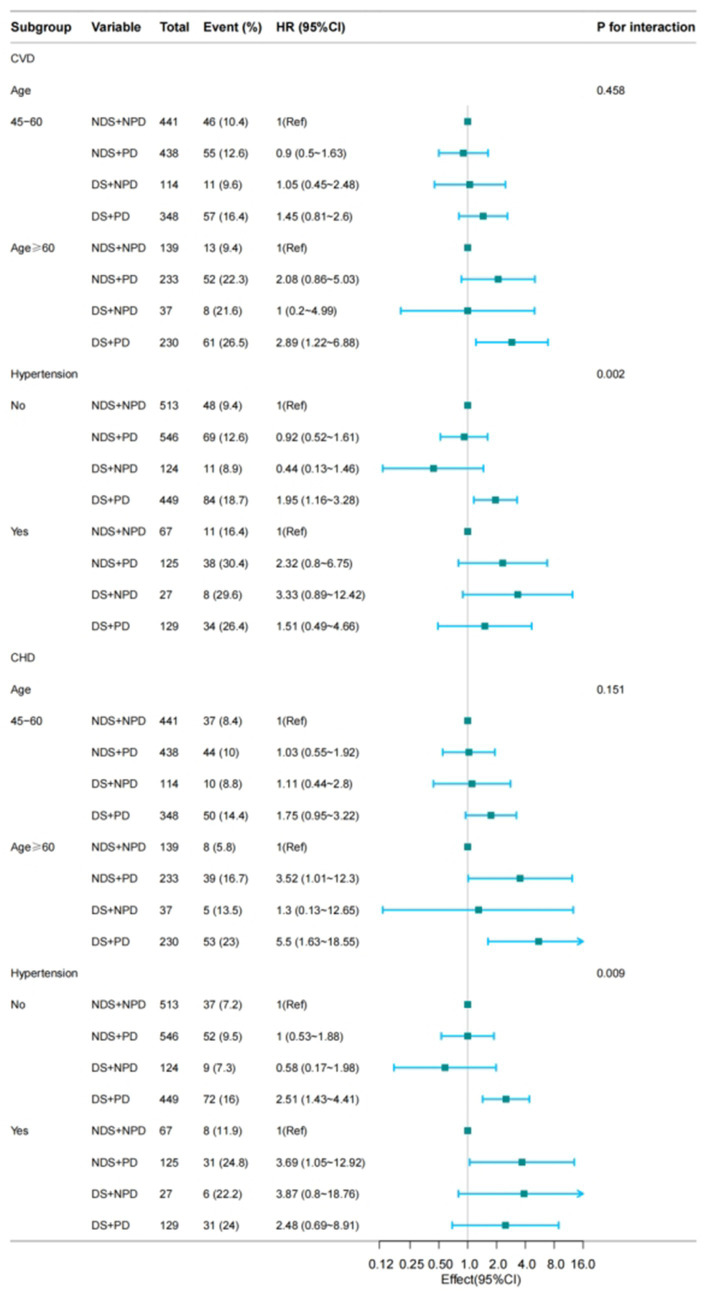
Subgroup analysis for the depressive symptoms, physical function, and on the risk of CVD (CVD, CHD). Model 3: Adjusted for hypertension (yes/no), dyslipidaemia (yes/no), diabetes mellitus (yes/no), and BUN (blood urea nitrogen), CREA (creatinine), Cys-C (cystatin-C), CHO (total cholesterol), HDL (High density lipoprotein cholesterol), LDL (Low Density Lipoprotein), TG (triglycerides), CRP (C-reaction protein), UA (Uric acid), HGB (hemoglobin), HbA1c (Glycated haemoglobins).

**Figure 3:**
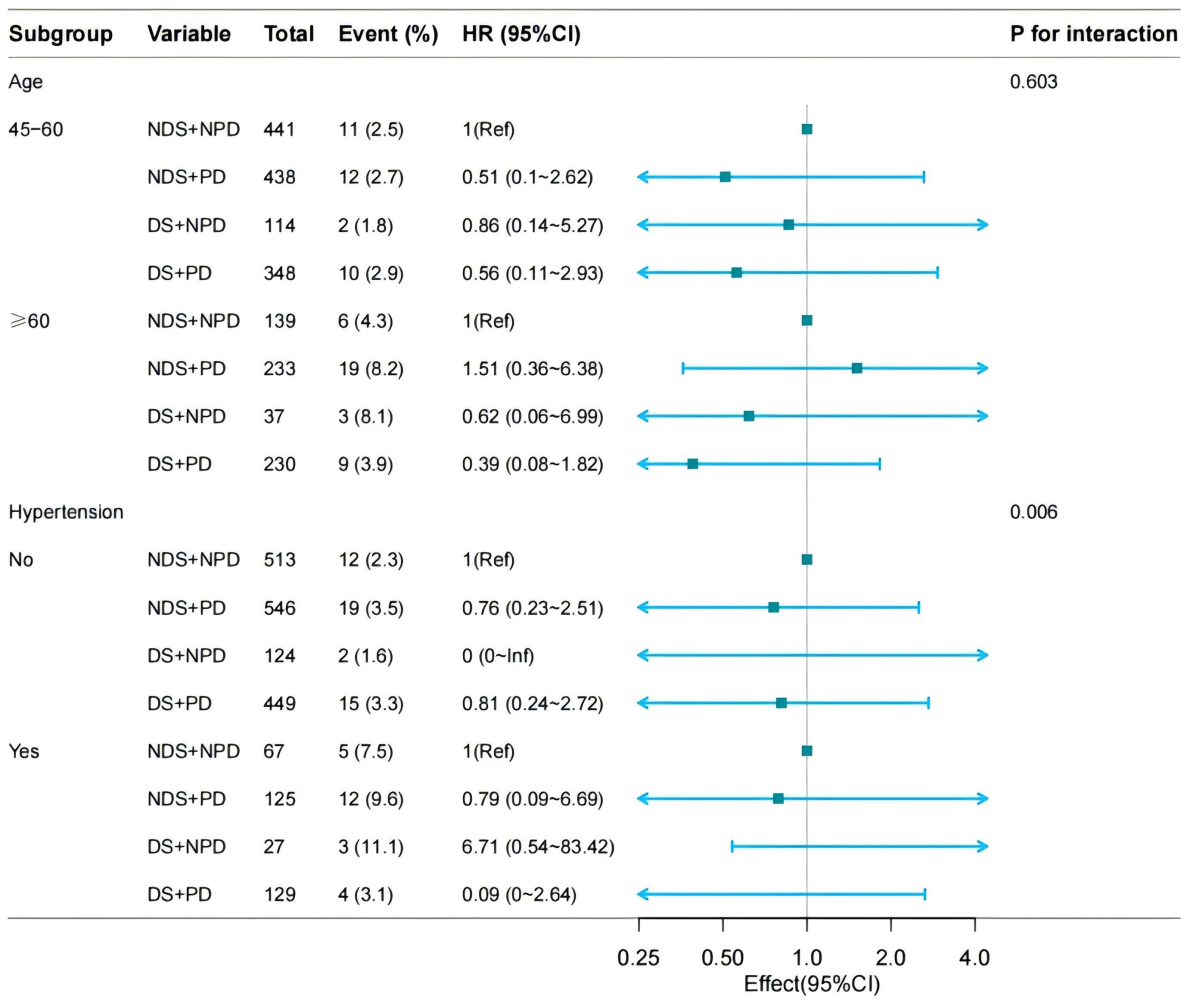
Subgroup analysis for the depressive symptoms, physical function, and on the risk of CVD (STOKE). Model 3: Adjusted for hypertension (yes/no), dyslipidaemia (yes/no), diabetes mellitus (yes/no), and BUN (blood urea nitrogen), CREA (creatinine), Cys-C (cystatin-C), CHO (total cholesterol), HDL (High density lipoprotein cholesterol), LDL (Low Density Lipoprotein), TG (triglycerides), CRP (C-reaction protein), UA (Uric acid), HGB (hemoglobin), HbA1c (Glycated haemoglobins).

In the Cox regression model, the reference groups were NDS + NPD group, In the subgroup of age strata (≥60 years) group with both DS and PD had a higher risk of CVD and CHD relative to the reference group [HR 2.89, 95% CI (1.22–6.88) HR 5.5, 95% CI (1.63–18.55)] (Figure 2). While The subgroup of hypertension (yes vs. no) showed a significant interaction for CVD (*p* for interaction = 0.002) and CHD (*p* for interaction = 0.009). In the non-hypertensive population, those with DS and PD had a higher risk of CVD and CHD [HR 1.95, 95% CI (1.16–3.28); HR 2.51, 95% CI (1.43–4.41)] (Figure 2). But in stroke, there was no significance [*p* for interaction = 0.013] (Figure 3; Table 6).

**Table 6:** Combined association of depressive symptoms and physical functioning with prevalence of CVD (interpolated).

Variables	Model 1		Model 2		Model 3	
	HR (95% CI)	adj. *p* value	HR (95% CI)	adj. *p* value	HR (95% CI)	adj. *p* value
CVD (cases/person-years)						
NDS + NPD (59/580)	1 (Ref)		1 (Ref)		1 (Ref)	
NDS + PD (107/671)	1.59 (1.17 ~ 2.15)	0.003	1.47 (1.06 ~ 2.04)	0.021	1.21 (0.75 ~ 1.94)	0.437
DS + NPD (19/151)	1.11 (0.67 ~ 1.83)	0.689	1.33 (0.79 ~ 2.24)	0.276	1.12 (0.53 ~ 2.4)	0.764
DS + PD (118/578)	2.06 (1.52 ~ 2.78)	<0.001	2.03 (1.46 ~ 2.82)	<0.001	1.87 (1.17 ~ 3)	0.009
CHD (cases/person-years)						
NDS + NPD (45/580)	1 (Ref)		1 (Ref)		1 (Ref)	
NDS + PD (83/671)	1.65 (1.17 ~ 2.34)	0.005	1.5 (1.03 ~ 2.17)	0.035	1.4 (0.92 ~ 2.14)	0.121
DS + NPD (15/151)	1.1 (0.61 ~ 1.95)	0.758	1.39 (0.77 ~ 2.5)	0.274	1.2 (0.59 ~ 2.43)	0.622
DS + PD (103/578)	2.34 (1.66 ~ 3.28)	<0.001	2.34 (1.62 ~ 3.39)	<0.001	2.13 (1.39 ~ 3.25)	<0.001
Stroke (cases/person-years)						
NDS + NPD (17/580)	1 (Ref)		1 (Ref)		1 (Ref)	
NDS + PD (31/671)	1.36 (0.77 ~ 2.41)	0.293	1.3 (0.71 ~ 2.41)	0.399	0.88 (0.41 ~ 1.9)	0.748
DS + NPD (5/151)	1.12 (0.45 ~ 2.81)	0.81	1.14 (0.42 ~ 3.1)	0.801	0.87 (0.24 ~ 3.17)	0.835
DS + PD (19/578)	1.11 (0.6 ~ 2.06)	0.73	0.93 (0.46 ~ 1.86)	0.831	0.63 (0.27 ~ 1.46)	0.277

Model 1: Adjusted for age (45–60/≥60), sex (male/female).

Model 2: Multivariate model adjusted for education (junior high school and below/senior high school and above), residence (urban/rural), smoking (yes/no), drinking (yes/no), BMI, marriage (yes/no), social activities (yes/no).

Model 3: Adjusted for hypertension (yes/no), dyslipidaemia (yes/no), diabetes mellitus (yes/no), and BUN (blood urea nitrogen), CREA (creatinine), Cys-C (Cystatin-C), CHO (total cholesterol), HDL (High density lipoprotein cholesterol), LDL (Low Density Lipoprotein), TG (triglycerides), CRP (C-reaction protein), UA (Uric acid), HGB (hemoglobin), HbA1c (Glycated haemoglobins).

### Sensitivity analyses

After interpolating missing data for sensitivity analyses, Comparison of DS, PD, and CVD between the non-interpolated population (*n* = 1980) and the interpolated population (*n* = 2,297) showed no difference in distribution (*p*>0.05) (Table 7). The association of DS and PD with Prevalence of CVD remained consistent among the interpolated data (2,297 participants) (Tables 5^6^).

**Table 7:** Comparison of exposure factors and endpoint events.

Variables	No missing variables	Insertion of BMI, education	adj. *p* value
DS	729/1980	875/2297	0.390
PD	1249/1980	1466/2297	0.616
CVD	303/1980	360/2297	0.739
CHD	246/1980	292/2297	0.777
Stroke	72/1980	86/2297	0.852

## Discussion

**Figure 4:**
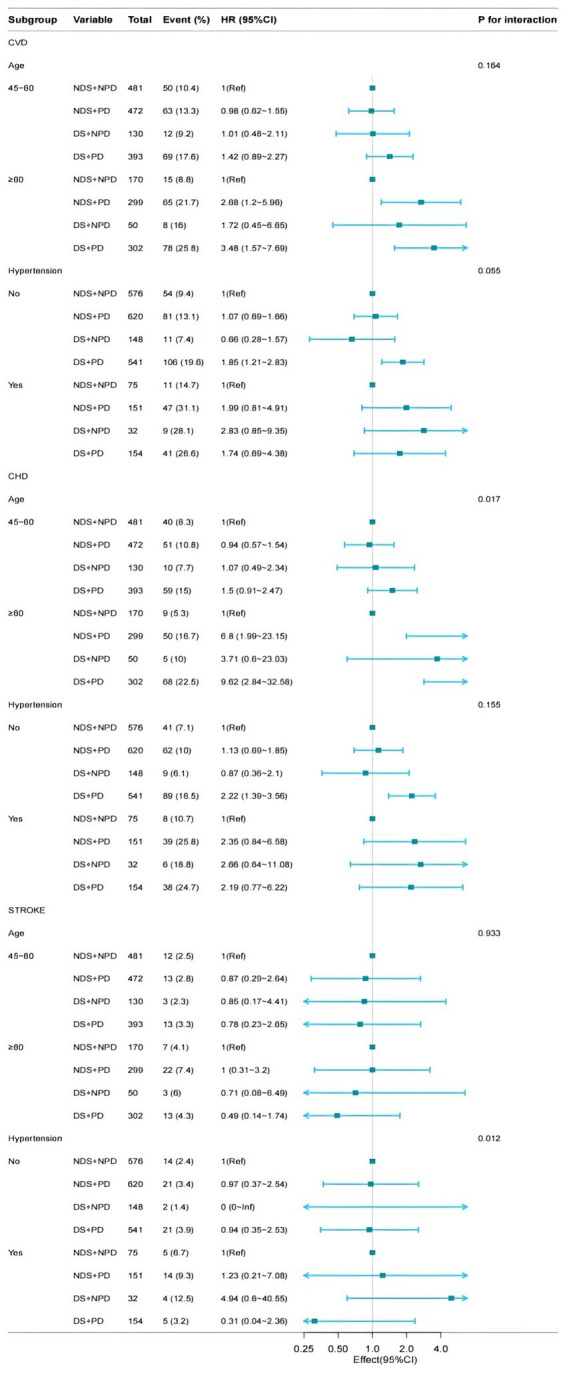
Subgroup analysis for the depressive symptoms, physical function, and on the risk of CVD (interpolated). Model 3: Adjusted for hypertension (yes/no), dyslipidaemia (yes/no), diabetes mellitus (yes/no), and BUN (blood urea nitrogen), CREA (creatinine), Cys-C (cystatin-C), CHO (total cholesterol), HDL (High density lipoprotein cholesterol), LDL (Low Density Lipoprotein), TG (triglycerides), CRP (C-reaction protein), UA (Uric acid), HGB (hemoglobin), HbA1c (Glycated haemoglobins).

Among 1980 Chinese adults aged 45 years or above followed up to 7 years, People with DS and PD was significantly associated with the highest risk of CVD events, especially among those aged 60 years or higher. The associations persisted even after adjustment for other established cardiovascular risk factors. Meanwhile, CVD was significantly increased in the population with concurrent DS and PD. In particular, the risk was significantly higher in the subgroup with PD (Figure 4).

A survey reveals that over 41% of senior Chinese citizens suffer from depression symptoms (13). Depressed patients commonly adopt habits that overlap with CV risk factors, such as sleep deprivation, physical inactivity, smoking and alcohol abuse, little hygiene and poor adherence to pharmacological treatments. A growing body of data suggests that depressive symptoms increase the risk of CVD (14). Recent studies have demonstrated a positive correlation between depressive symptoms and the risk of cardiovascular disease in young adults (mean age 30.1 years) (12). Current research suggests that depression may make CVD more common in women (13). Additionally, it has been shown that a significant risk factor for the emergence of depressive symptoms is physical dysfunction (5). Poorer mental health is associated with lower exercise capacity, above and beyond the effect of other cardiovascular risk factors (15). At the same time, a decline in physical function leads to a loss of independence and consequent depressive symptoms. These studies demonstrated that those with the lowest levels of physical function carry the largest risk of onset of both depressive symptoms over time (16). Recent studies have shown the association between physical dysfunction and cardiovascular disease risk (6, 7, 17, 18). PD can be assessed by more aspects, but the above studies preferred the assessment of upper limb strength, our study included not only upper limb strength but also lower limb strength and physical activities, which was more comprehensive.

The mechanism between DS and CVD risk factors remains unclear. However, Previous studies showed that DS were associated with unhealthy behaviors that promoted weight gain, such as consumption of a high-fat diet and low levels of physical activity (16). Similarly, depression is associated with higher rates of smoking, another risk factor for hypertension, and diabetes (19). Inflammatory markers have been consistently linked to elevated DS, and are closely associated with obesity, hypertension, and diabetes. Other candidate biological pathways that may be responsible for these associations include pro-coagulant factors (20), sympathetic activation (21), endothelial dysfunction (21), and adipocytokines, such as leptin (22), this ultimately leads to CVD.

The burden of DS in China has been rising and will continue to climb in the next decades (23). Studies have shown that declining physical functioning in older adults is a major risk factor for developing DS (24). In addition, the prevalence rates of depression in Chinese older adults ranged from 13 to 41% and is on the rise, with further growth expected in the following decades (3, 25). However reduced physical mobility will inevitably lead to PD, along with upregulation of inflammatory factors and accelerated endothelial dysfunction, leading to CVD (15). Therefore, it is necessary to explore the relationship between DS combined with PD and CVD risk.

We discovered that those with DS + PD were significantly associated with higher CVD, although it was not significant for those with DS alone. The impact of PD on CVD varies across populations and more data are needed to investigate this. In the present study, we found a higher risk of CVD in people ≥60 years of group DS + PD, consistent with a cross-sectional study in the UK (8). In people without hypertension, patients with DS and PD are at higher risk of developing CVD. In stroke, however, there was no significant relationship in the COX multifactorial analysis and subgroup analyses after model adjustment. This may be related to the small number of stroke positives we included, and further expansion of the sample size is needed to explore the association between DS, PD, and stroke. The risk of cardiovascular disease and coronary heart disease was not statistically significant in the NDS + PD and DS + NPD groups compared with the NDS + NPD group. Therefore, we should pay more attention to elderly people with DS and PD when assessing CVD risk.

### Strengths and limitations

The strengths of our study are, Data on DS and PD were taken from the CHARLS database, a sizable prospective cohort with carefully thought out and verified procedures. Ability to better infer the triad of DS, PD and CVD. We additionally took a number of confounders into account, such as BMI and social activity. Our application of the questionnaire from the CHARLS database allowed us to assess PD in a comprehensive way. Notably, this is the first time that the relationship between DS, PD and CVD has been explored in an Asian elderly population. However, there are certain limits. Firstly, the CESD-10 may exhibit recall bias and should only be used to screen for depressive symptoms, not to diagnose depression. Secondly, in stroke, however, there was no significant relationship in the COX multifactorial analysis and subgroup analyses after model adjustment. This may be related to the small number of stroke positives we included, and further expansion of the sample size is needed to explore the association between DS, PD, and stroke. Third, reliance on patient-reported diagnoses (e.g., hypertension, diabetes) may introduce recall bias or misclassification. Although self-reported CVD risk factors are widely used in large cohort studies (26), future studies should incorporate objective biomarkers to improve accuracy.

## Conclusion

In conclusion, through a prospective national cohort study of Chinese middle-aged and elderly adults, we found that the associations of depressive symptoms, physical functioning and CVD risk were significantly related. The findings highlight the co-exposure effect of depressive symptoms and physical functioning in terms of incident CVD and suggest a joint assessment of depressive symptoms and physical functioning to further stratify CVD risk, especially in those adults≥60 years and without hypertension. This should be considered as a potential modifying factor in clinical trials for interventions in CVD research.

## Data Availability

Publicly available datasets were analyzed in this study. This data can be found at: http://charls.pku.edu.cn.

## Footnotes

^1^ https://www.clinicalscientists.cn/freestatistics/

## References

[1] Roth GA Mensah GA Johnson CO Addolorato G Ammirati E Baddour LM. Global burden of cardiovascular diseases and risk factors, 1990-2019: update from the GBD 2019 study. J Am Coll Cardiol. (2020) 76:2982–3021. doi: 10.1016/j.jacc.2020.11.010, PMID: 33309175 PMC7755038

[2] Villas-Boas SB White JS Kaplan S Hsia RY. Trends in depression risk before and during the COVID-19 pandemic. PLoS One. (2023) 18:e0285282. doi: 10.1371/journal.pone.0285282, PMID: 37195921 PMC10191294

[3] Lu J Xu X Huang Y Li T Ma C Xu G. Prevalence of depressive disorders and treatment in China: a cross-sectional epidemiological study. Lancet Psychiatry. (2021) 8:981–90. doi: 10.1016/S2215-0366(21)00251-0, PMID: 34559991

[4] Harshfield EL Pennells L Schwartz JE Willeit P Kaptoge S Bell S. Association between depressive symptoms and incident cardiovascular diseases. JAMA. (2020) 324:2396–405. doi: 10.1001/jama.2020.23068, PMID: 33320224 PMC7739139

[5] Yan Y Du Y Li X Ping W Chang Y. Physical function, ADL, and depressive symptoms in Chinese elderly: evidence from the CHARLS. Front Public Health. (2023) 11:1017689. doi: 10.3389/fpubh.2023.1017689, PMID: 36923048 PMC10010774

[6] Ramakrishnan R Doherty A Smith-Byrne K. Accelerometer measured physical activity and the incidence of cardiovascular disease: evidence from the UK biobank cohort study. PLoS Med. (2021) 18:e1003809. doi: 10.1371/journal.pmed.1003809 33434193 PMC7802951

[7] Zengin A Ó Breasail M Parsons CM. Sex-specific associations between cardiovascular risk factors and physical function: the Gambian bone and muscle ageing study. J Cachexia Sarcopenia Muscle. (2023) 14:84–92. doi: 10.1002/jcsm.13069, PMID: 36346161 PMC9891990

[8] Ludwig VM Bayley A Cook DG Stahl D Treasure JL Asthworth M. Association between depressive symptoms and objectively measured daily step count in individuals at high risk of cardiovascular disease in South London, UK: a cross-sectional study. BMJ Open. (2018) 8:e020942. doi: 10.1136/bmjopen-2017-020942, PMID: 29654044 PMC5898324

[9] Chen H Mui AC. Factorial validity of the Center for Epidemiologic Studies Depression Scale short form in older population in China. Int Psychogeriatr. (2014) 26:49–57. doi: 10.1017/S1041610213001701, PMID: 24125553

[10] Boey KW. Cross-validation of a short form of the CES-D in Chinese elderly. Int J Geriatr Psychiatry. (1999) 14:608–17. doi: 10.1002/(SICI)1099-1166(199908)14:8<608::AID-GPS991>3.0.CO;2-Z, PMID: 10489651

[11] Mengqi L. Research on the influencing factors of mental health and policy suggestions for the elderly in China. Zhejiang: Zhejiang University (2018).

[12] Chirinos DA Kershaw KN Allen NB Carroll AJ Lewis TT Schreiner PJ. Depressive symptom subgroups and their association with prevalent and incident cardiovascular risk factors in the coronary artery risk development in young adults (CARDIA) study. Int J Behav Med. (2023) 30:891–903. doi: 10.1007/s12529-022-10144-z, PMID: 36670342

[13] Bucciarelli V Caterino AL Bianco F Caputi CG Salerni S Sciomer S. Depression and cardiovascular disease: the deep blue sea of women's heart. Trends Cardiovasc Med. (2020) 30:170–6. doi: 10.1016/j.tcm.2019.05.001, PMID: 31109802

[14] Goldstein BI Carnethon MR Matthews KA McIntyre R Miller GE Raghuveer G. Major depressive disorder and bipolar disorder predispose youth to accelerated atherosclerosis and early cardiovascular disease: a scientific statement from the American Heart Association. Circulation. (2015) 132:965–86. doi: 10.1161/CIR.0000000000000229, PMID: 26260736

[15] Lv S Shi Y Xue Y Hu Y Hu M Li S. Long-term effects of particulate matter on incident cardiovascular diseases in middle-aged and elder adults: the CHARLS cohort study. Ecotoxicol Environ Saf. (2023) 262:115181. doi: 10.1016/j.ecoenv.2023.115181, PMID: 37393817

[16] Räikkönen K Matthews KA Kuller LH. Depressive symptoms and stressful life events predict metabolic syndrome among middle-aged women: a comparison of World Health Organization, adult treatment panel III, and international diabetes foundation definitions. Diabetes Care. (2007) 30:872–7. doi: 10.2337/dc06-1857 17392548

[17] Bin Sayeed MS Joshy G Paige E Banks E Korda R. Cardiovascular disease subtypes, physical disability and workforce participation: a cross-sectional study of 163,562 middle-aged Australians. PLoS One. (2021) 16:e0249738. doi: 10.1371/journal.pone.0249738, PMID: 33831054 PMC8031377

[18] German C Makarem N Fanning J. Sleep, sedentary behavior, physical activity, and cardiovascular health: MESA. Med Sci Sports Exerc. (2021) 53:724–31. doi: 10.1249/MSS.0000000000002534, PMID: 33044436 PMC7969364

[19] Brummett BH Babyak MA Siegler IC Mark DB Williams RB Barefoot JC. Effect of smoking and sedentary behavior on the association between depressive symptoms and mortality from coronary heart disease. Am J Cardiol. (2003) 92:529–32. doi: 10.1016/S0002-9149(03)00719-7, PMID: 12943871

[20] Strike PC Steptoe A. Psychosocial factors in the development of coronary artery disease. Prog Cardiovasc Dis. (2004) 46:337–47. doi: 10.1016/j.pcad.2003.09.001, PMID: 14961456

[21] Licht CM de Geus EJ van Dyck R Penninx BW. Association between anxiety disorders and heart rate variability in the Netherlands study of depression and anxiety (NESDA). Psychosom Med. (2009) 71:508–18. doi: 10.1097/PSY.0b013e3181a292a6, PMID: 19414616

[22] Chirinos DA Goldberg R Gellman M Mendez AJ Gutt M McCalla JR. Leptin and its association with somatic depressive symptoms in patients with the metabolic syndrome. Ann Behav Med. (2013) 46:31–9. doi: 10.1007/s12160-013-9479-5, PMID: 23436275 PMC3696025

[23] Charlson FJ Baxter AJ Cheng HG Shidhaye R Whiteford HA. The burden of mental, neurological, and substance use disorders in China and India: a systematic analysis of community representative epidemiological studies. Lancet. (2016) 388:376–89. doi: 10.1016/S0140-6736(16)30590-6, PMID: 27209143

[24] Kvæl LAH Bergland A Telenius EW. Associations between physical function and depression in nursing home residents with mild and moderate dementia: a cross-sectional study. BMJ Open. (2017) 7:e016875. doi: 10.1136/bmjopen-2017-016875, PMID: 28729326 PMC5541615

[25] Stegenga BT Nazareth I Torres-González F Xavier M Švab I Geerlings MI. Depression, anxiety and physical function: exploring the strength of causality. J Epidemiol Community Health. (2012) 66:e25. doi: 10.1136/jech.2010.128371 21693471

[26] Cui H Liu Q Wu R Cao L. Cumulative triglyceride-glucose index is a risk for CVD: a prospective cohort study. Cardiovasc Diabetol. (2022) 21:21 doi: 10.1186/s12933-022-01456-1 35144621 PMC8830002

